# Real-time dataset of pond water for fish farming using IoT devices

**DOI:** 10.1016/j.dib.2023.109761

**Published:** 2023-11-04

**Authors:** Md. Monirul Islam

**Affiliations:** Department of Software Engineering, Daffodil International University, Daffodil Smart City (DSC), Birulia, Savar, Dhaka 1216, Bangladesh

**Keywords:** Smart fish farming, Aquatic biology, IoT sensor

## Abstract

This paper introduces a real-time water quality dataset of five ponds for fish farming obtained through an IoT framework for monitoring the aquatic environmental conditions. It utilizes sensors and an Arduino microcontroller to collect data on pH, temperature, and turbidity in pond water in Jamalpur District, Bangladesh. The data is stored in an IoT cloud platform named ThingSpeak and analyzed using 10 machine learning algorithms. The dataset consists of 4 columns and 40,280 rows, where pH, temperature, turbidity, and fish are recorded. Fish represents the target variable, while the others serve as independent variables. Within the dataset, there are 11 distinct fish categories including sing, silver carp, Katla, prawn, karpio, shrimp, rui, pangas, tilapia, magur, and koi. Results showed that only three ponds are suitable for fish farming among five ponds and the Random Forest algorithm performs the best. The study also includes details of the IoT system's hardware. This dataset will be useful for researchers and fish farmers to predict fish survival.

Specifications Table

**Table:** 

Subject	Internet of Things (IoT), Machine Learning and Aquaculture
Specific subject area	Aquatic Environment Monitoring, IoT devices for fish farming, Smart Agriculture, Machine Learning.
Type of data	Comma Separated Value (CSV)
How the data were acquired	An IoT Framework; An IoT framework has been created for monitoring the aquatic environmental parameters concerning water quality such as Ph, Temperature as well as turbidity. The framework operates as an embedded system, employing sensors and an Arduino platform. In pond water used for cultivation, several sensors, such as pH, temperature, and turbidity sensors, are strategically placed, each connected to a shared microcontroller based on the Arduino Uno. These sensors retrieve data from the water and save it in a comma-separated values (CSV) format within an IoT cloud platform called ThingSpeak, facilitated by the Arduino microcontroller. To validate the experiment's effectiveness, data was collected from five ponds of varying sizes and environmental conditions.
Data format	Raw Analyzed Filtered
Description of data collection	The dataset consists of 4 columns and 40280 rows, where pH, temperature, turbidity, and fish are recorded. Fish represents the target variable, while the others serve as independent variables. Within the dataset, there are 11 distinct fish categories including sing, silver carp, Katla, prawn, karpio, shrimp, rui, pangas, tilapia, magur, and koi, having distinct values of tilapia 8830 rui 6336 pangas 5314 silverCup 3906 katla 3786 sing 3776 shrimp 3204 karpio 2112 prawn 1348 koi 964 magur 704.
Data source location	Institution: Department of Software Engineering, Daffodil International University, Daffodil Smart City (DSC), Birulia, Savar, Dhaka 1216, Bangladesh.
Data accessibility	Repository name: Mendeley Data Data identification number: DOI:10.17632/hxd382z2fg.2 Direct URL to data: https://data.mendeley.com/datasets/hxd382z2fg/2[6]
Related research article	Islam M.M., Kashem M.A., Alyami S.A., Moni M.A., Monitoring water quality metrics of ponds with IoT sensors and machine learning to predict fish species survival, Microprocessors and Microsystems (2023): https://www.sciencedirect.com/science/article/abs/pii/S0141933123001746

## 1 Value of the Data

• These data are useful because they will be used to research in the aquaculture fields to predict the survival of fish species.
• The fish farmer will greatly benefit as they can take decisions which they have to make for fish production.
• These data will be used by researchers to predict the survival of fish specifies automatic sign language words recognition.
• The researcher of aquaculture can know the standard values of all water quality index and as per this, they can be influenced to do research for further experiments to get more values.
• The dataset is essential for newcomers in machine learning or data science to insight the data.
• This dataset will greatly contribute to the researchers and fish farmers.

## 2 Objective

The main objective behind the generation of the dataset is to check the ponds' water quality for fish cultivation. Different fish survive in different parameter levels of water like temperature, Ph and turbidity. That is why, the author created an IoT framework to read the real-time data of aquatic environment. After the literature study of life cycle of 11 different types of fishes, author compared the real-time data with the standard values (pH (6.5–8.5), temperature (16–24 °C), turbidity (below 10 ntu)). After that, author concluded that 3 ponds were perfect for fish farming among the 5 ponds. In addition, to validate the data, author used machine learning models to analysis the data. By using this IoT system, the farmers will be benefitted to take the proper decision for more production and less death rate of fishes. Moreover, the researchers will be helpful to contribute more to establish the system to get long time real values. The dataset presented here complements an original research paper that has been accepted and is currently in press. Therefore, this data article enhances the value of the published research by providing a clear demonstration of the dataset's functionality and its effectiveness in analyzing real-time aquatic data. It aims to offer readers a comprehensive understanding of the dataset, facilitating easy comprehension and potential reuse for further research in the field of fish farming.

## 3 Data Description

This dataset represents real-time information collected from a monitoring system designed to assess aquatic conditions using an IoT framework. The system employs three sensors - pH, temperature, and turbidity, in conjunction with an Arduino controller, to continuously evaluate water quality in five distinct ponds. The dataset comprises 40,280 records and contains four columns: pH, temperature, turbidity, and fish. Among these columns, “fish” serves as the target variable, while the remaining three are considered independent variables. Within the dataset, there are eleven distinct fish categories, having distinct values of tilapia 8830 rui 6336 pangas 5314 silverCup 3906 katla 3786 sing 3776 shrimp 3204 karpio 2112 prawn 1348 koi 964 magur 704. The dataset still possesses certain limitations, including its dataset size. A research paper has been published against this dataset [1]. The dataset size can be extended using the premium cloud protocol and for a long time run of the system. Table 1 shows some data as a sample.

**Table 1:** Some data as sample.

pH	Temperature	Turbidity	Fish
6	27	4	katla
7.6	28	5.9	sing
7.8	27	5.5	sing
6.5	31	5.5	katla
8.4	25	5	rui
6.1	31	4.9	rui
5.5	18	5	koi
7.1	23	5.5	koi
7.5	29	6	prawn
7.9	29	7.2	prawn
7.8	27	5.6	sing
8	27	3.8	pangas
6.5	21	5	tilapia
5.9	25	5.5	tilapia

**Fig. 1:**
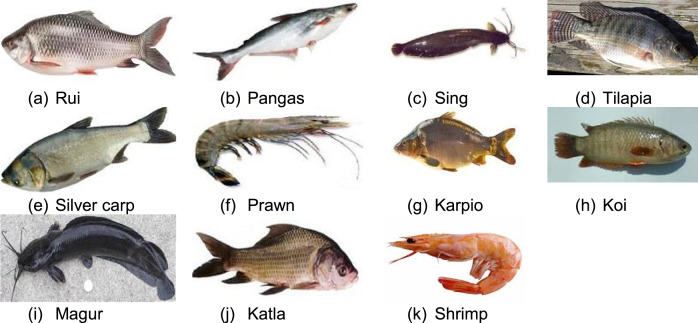
11 fishes.

**Fig. 2:**
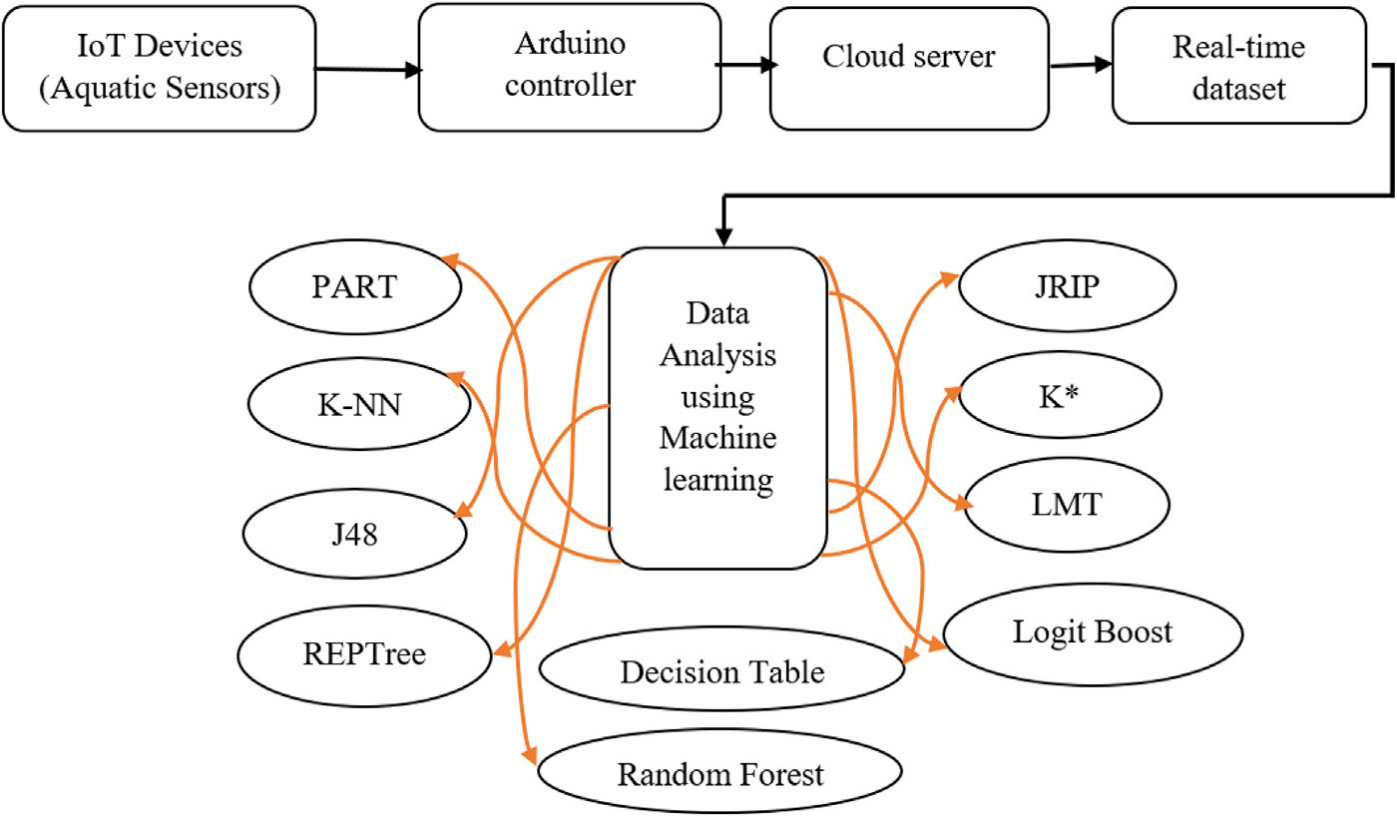
Workflow of designed method.

Fig. 2 represents 11 fishes of which lies with our dataset. After getting the dataset, we labeled the target variable as fish as per the standard values of aquatic environment of each fish. The image of 11 fishes is shown in Fig. 1.

## 4 System Design and Methods

The overall proposed method of getting the real-time aquatic values from the IoT framework is shown in Fig. 2. First of all, we defined the aquatic sensors then the Arduino setup read the data through the sensors and stored the data in cloud protocol. From the cloud, we downloaded the data then labelling with the fishes. At the final stage, we divided the work into two parts named pond selection based on the reference values and another is prediction survival of fish specifies.

**Fig. 3:**
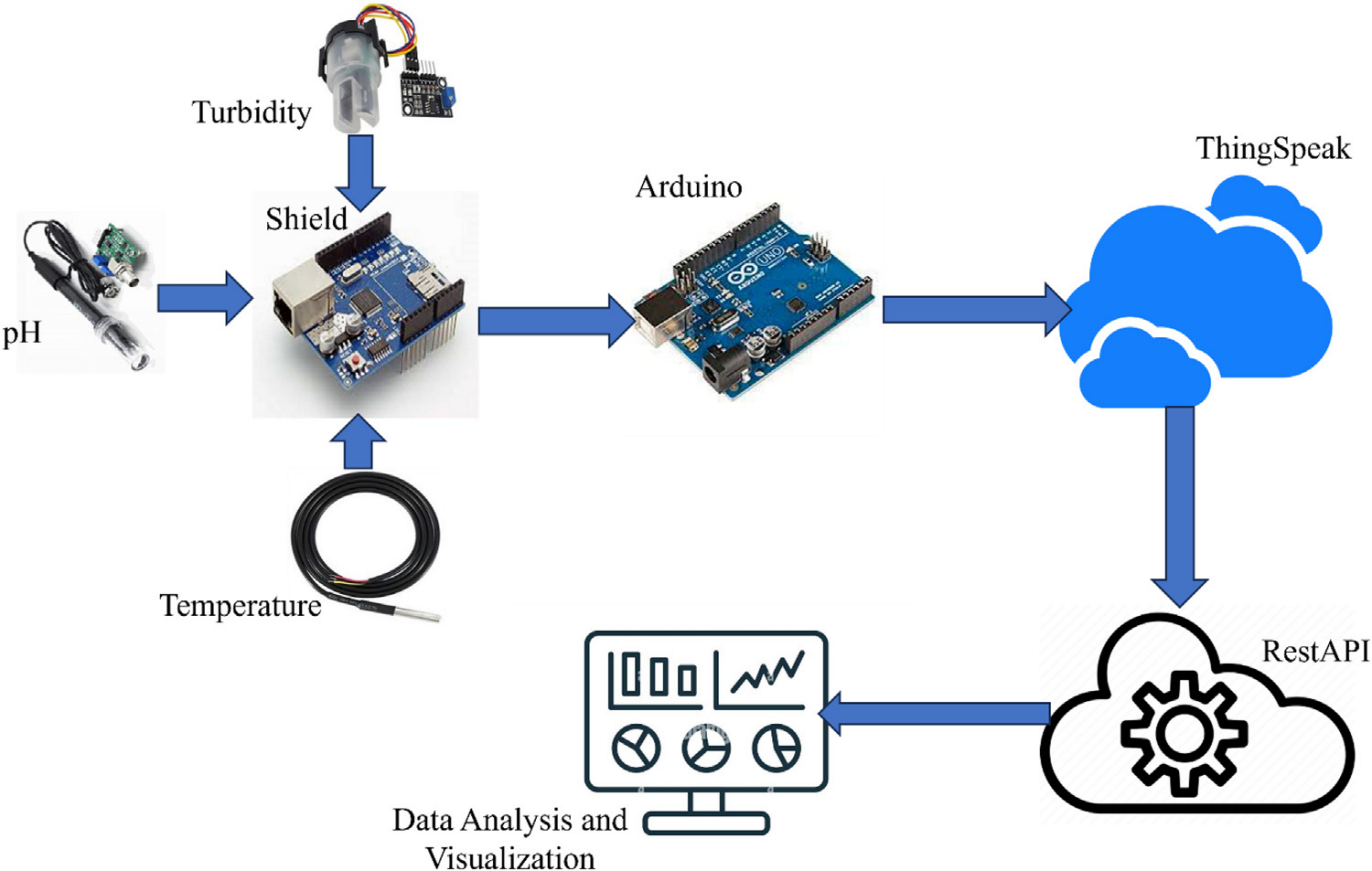
Block diagram of proposed methodology.

The dataset with the fish specifies is evaluated by machine learning models to predict the fish survival [2]. The physical part of the IoT framework is displayed in Fig. 3. Several researches have been done in this field like [3], [4], [5].

**Fig. 4:**
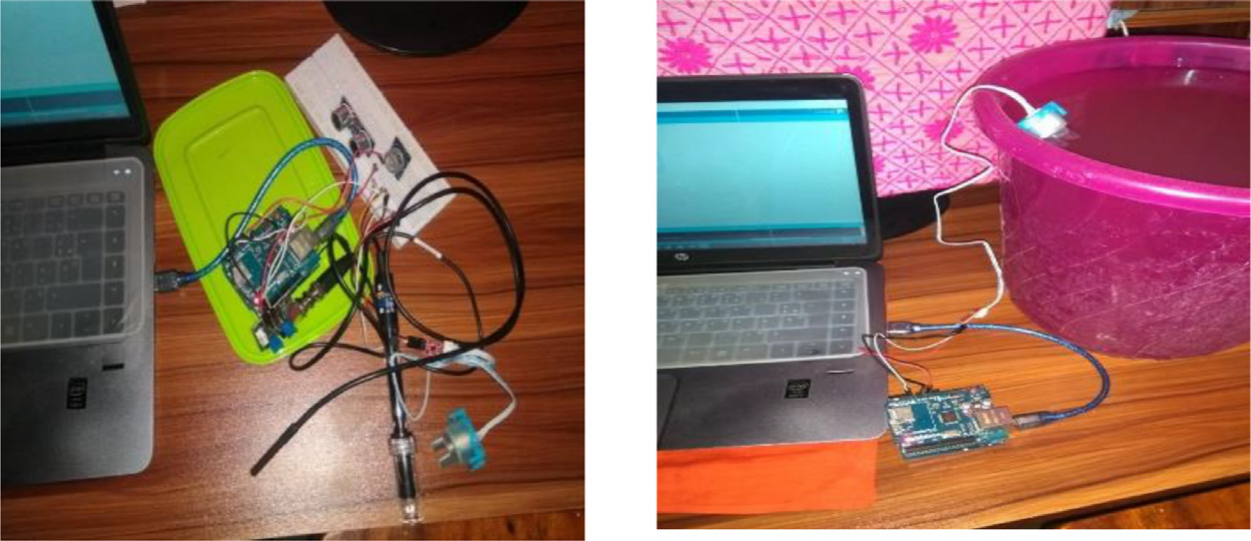
Some experimental images.

Fig. 4 shows some of the experimental images of the proposed system.

## 5 Pond Suggestion

We identified ponds suitable for fish farming based on specific criteria, including pH levels within the range of 6.5–8.5, turbidity below 10 ntu, a temperature range of 16–24 °C, and pond depth within the range of 1–5 m, with an optimal depth of 2 m for fish farming. We assessed the pond depths manually using a measuring stick. Table 2 provides an overview of the real-time data collected for each pond, summarizing their compliance with these criteria.

**Table 2:** Received values for suggesting pond where Temp = temperature, Tur = Turbidity.

Pond No	pH	Temp (°C)	Tur (ntu)	Depth (m)	Remarks
01	6.02–8.39	17.50–17.75	3.55–3.57	1–2	Suggested
02	8.57–8.87	17.75–18.00	3.41–3.50	1–2	Not suggested
03	6.00–7.83	20.87–21.06	3.31–3.49	1–2	Suggested
04	6.51–8.30	21.06–21.44	3.60–3.62	2–4	Suggested
05	3.84–3.95	21.06–21.25	3.56–3.58	1–3	Not suggested

Based on this data, it is recommended to consider pond-1, pond-3, and pond-4 for fish farming purposes. However, pond-2 is not suitable due to its pH levels, which range between 8.57 and 8.87, exceeding the ideal range and potentially hindering fish growth. Pond-5 is also unsuitable due to its low pH value of 3.84–3.95, which can lead to fish mortality. Consequently, we do not recommend ponds 1 and 2 for fish farming.

## 6 Comparison Performance Metrics Based on Machine Learning

Real-time data values are subjected to analysis through the utilization of ten distinct machine learning (ML) algorithms. Table 3 presents a comparative assessment of performance metrics, encompassing accuracy, kappa statistics (KS), as well as the average true positive rate, across these classifiers.

**Table 3:** Comparison among classification model based on the performance metric.

ML model	Accuracy	KS	Avg. TP rate	Place
PART	90.35 %	88.92 %	90.4 %	4th
Decision table	80.54 %	77.5 %	80.5 %	**10**th
K-NN	93.4 %	92.4 %	93.4 %	2nd
K* Algorithm	89.85 %	88.37 %	89.8 %	6th
J48	90.19 %	88.7 %	90.2 %	5th
Random forest	94.42 %	93.5 %	94.4 %	**1**st
JRIP	87.14 %	85.17 %	87.1 %	7th
Logit boost	84.60 %	82.37 %	84.6 %	8th
LMT	92.22 %	91.08 %	92.2 %	3rd
REPTree	83.93 %	81.5 %	83.9 %	9th

Table 3 presents a comprehensive breakdown of performance metrics across various machine learning algorithms. Notably, Random Forest stands out as the top performer, achieving remarkable scores in accuracy (94.42 %), kappa statistic (93.51 %), and the average True Positive (TP) rate (94.4 %). Following closely, the KNN model secures the second-highest rankings with accuracy at 93.4 %, KS at 92.4 %, as well as a TP rate of 93.4 %. In the third position, the LMT model attains an accuracy of 92.22 %, a KS of 91.08 %, and a TP rate of 92.2 %. PART occupies the fourth spot, displaying an accuracy of 90.35 %, a kappa statistic of 88.92 %, and a TP rate of 90.4 %. The fifth position goes to J48, recording an accuracy of 90.195 %, a kappa statistic of 88.7 %, and an average TP rate of 90.2 %. K* ranks sixth across all metrics with an accuracy score of 89.85 %, a KS of 88.37 %, as well as an average True Positive rate of 89.8 %. The seventh spot is claimed by the JRIP model, with an accuracy of 87.14 %, a KS of 85.17 %, and an average TP rate of 87.1 %. Logit Boost secures the eighth position, with an accuracy of 84.60 %, a kappa statistic of 82.37 %, and a TP rate of 84.6 %. REPTree follows in ninth place, registering an accuracy of 83.93 %, a KS of 81.5 %, and a TP rate of 83.9 %. Lastly, the decision table ranks lowest across all metrics, with an accuracy of 80.54 %, a kappa statistic of 77.5 %, and an average TP rate of 80.5 %.

Random Forest emerges as the top performer across all metrics, owing to its ensemble learning nature, where decisions are made by aggregating votes from multiple decision trees within the algorithm. This democratic approach results in highly accurate and reliable predictions.

## Ethics Statements

The experiment was done to collect data from Malancha village under Melandah Upazilla of Jamalpur District, Bangladesh.

## CRediT authorship contribution statement

**Md. Monirul Islam:** Conceptualization, Methodology, Investigation, Formal analysis, Writing – original draft, Writing – review & editing.

## Data Availability

A Real-Time Dataset of Pond Water for Fish Farming using IoT devices (Original data) (Mendeley Data)
